# Effect of Silk Fibroin on Cell Viability in Electrospun Scaffolds of Polyethylene Oxide

**DOI:** 10.3390/polym11030451

**Published:** 2019-03-09

**Authors:** Gabriela Carrasco-Torres, Manuel A. Valdés-Madrigal, Verónica R. Vásquez-Garzón, Rafael Baltiérrez-Hoyos, Eduard De la Cruz-Burelo, Ramón Román-Doval, Anaí A. Valencia-Lazcano

**Affiliations:** 1 Departamento de Nanociencias y Nanotecnología. Centro de Investigación y de Estudios Avanzados del IPN. Av. IPN 2508, la laguna Ticomán, Ciudad de México 07360, Mexico; carrasco@cell.cinvestav.mx (G.C.-T.); mavm8405@hotmail.com (M.A.V.-M.); 2 Instituto Tecnológico Superior de Ciudad Hidalgo. Av. Ing. Carlos Rojas Gutiérrez 2120, fracc. Valle de la herradura, Michoacán 61100, Mexico; 3 CONACYT-Facultad de Medicina y Cirugía Universidad Autónoma Benito Juárez de Oaxaca, Ex Hacienda de Aguilera S/N, Carretera a San Felipe del Agua S/N, Oaxaca 68020, Mexico; vrvasquezga@conacyt.mx (V.R.V.-G.); rbaltierrezho@conacyt.mx (R.B.-H.); 4 Centro de Investigación y de Estudios Avanzados del IPN, Av. IPN 2508, la laguna Ticomán, Ciudad de México 07360, Mexico; e.delacruz.burelo@cinvestav.mx; 5 Departamento de investigación y posgrado en alimentos, Facultad de Química, Universidad Autónoma de Querétaro, Centro universitario, Santiago de Querétaro, Querétaro 76010, Mexico

**Keywords:** silk fibroin, surface modification, electrospinning, ultrafiltration

## Abstract

In this study, a coating from electrospun silk fibroin was performed with the aim to modify the surface of breast implants. We evaluated the effect of fibroin on polymeric matrices of poly (ethylene oxide) (PEO) to enhance cell viability, adhesion, and proliferation of HaCaT human keratinocytes to enhance the healing process on breast prosthesis implantation. We electrospun six blends of fibroin and PEO at different concentrations. These scaffolds were characterized by scanning electron microscopy, contact angle measurements, ATR-FTIR spectroscopy, and X-ray diffraction. We obtained diverse network conformations at different combinations to examine the regulation of cell adhesion and proliferation by modifying the microstructure of the matrix to be applied as a potential scaffold for coating breast implants. The key contribution of this work is the solution it provides to enhance the healing process on prosthesis implantation considering that the use of these PEO–fibroin scaffolds reduced (p < 0.05) the amount of pyknotic nuclei. Therefore, viability of HaCaT human keratinocytes on PEO–fibroin matrices was significantly improved (p < 0.001). These findings provide a rational strategy to coat breast implants improving biocompatibility.

## 1. Introduction

The modification of fibers has been used successfully to improve diverse applications, such as mimicking the properties of extracellular matrices, incorporating nanomaterials for biosensors, or to give specific characteristic to improve electrical and mechanical properties [1,2,3]. Surface modification has the purpose of enhancing the surface of a material by modifying its original chemical, physical, or biological surface characteristics [4,5]. The surface of many materials may be tailored to improve their properties such as topography, chemistry, and wettability to create a micro-environment that influences protein adsorption, cell adhesion, and bioactivity [6,7].

Breast implants should interact with the tissue to create an appropriate bond and initiate the wound-healing process [8]. Epithelialization is an essential piece of wound healing. Keratinocytes are the major cellular component of the epidermis and are responsible for restoring the epidermis after injury. Keratinocytes start to proliferate so that a suitable supply of cells encloses the wound. Proliferation of keratinocytes depends on the degree of cell differentiation, availability of growth factors, and cell attachment to the substrate [9]. Specific designs by polymer processing technologies in combination with surface coating and chemical modification enable the interaction of medical devices with tissue to create a structural microenvironment for cells during scaffold seeding and tissue formation [10,11].

Electrospinning is technique used to create micro and nanostructures that serve as microenvironments for cells to adhere and proliferate. A synthetic polymer used to create electrospun mats is poly (ethylene oxide) (PEO), which has been used extensively in the biomedical applications due to its non-toxicity, its ability to dissolve in aqueous solutions and organic solvents, and its uncomplicated elimination by the renal and hepatic pathways. Moreover, PEO can be used as support material to allow electrospinning of other materials and enhance the fiber functionalities which inside the human body have demonstrated to be nearly chemically inert [12,13]. Moreover, PEO solutions have been used to electrospin polymer fibers of 50–750 nm diameter, while collagen fibrils of 100–500 nm are found in the native extracellular matrix, resulting in highly effective cell guidance applications [14]. In addition, PEO has shown high versatility as a carrier polymer. Therefore, a combination of controlled fiber diameters and specific protein loading such as silk fibroin may support tissue regeneration [15]. Building upon evidence that silk fibroin promotes tissue regeneration, recent approaches have studied its potential as a coating for implants [16]. It has been shown that coating an implant with silk fibroin makes it more biocompatible by improving cell attachment and reducing bacterial infection on implants [17].

We extracted fibroin from *Bombyx mori* cocoon shells to be desalted by ultrafiltration to test its efficacy. The benefit of creating these polymeric matrices of PEO-supported silk fibroin is that they are expected to coat effectively the surfaces of breast implants in order to enhance cell viability, adhesion, and proliferation of HaCaT human keratinocytes and enhance the healing process. Therefore, we choose to electrospin six blends of fibroin and PEO at different concentrations for culturing HaCaT cells. We examined the morphology effect of the scaffolds on cell viability to be applied as a potential scaffold for breast implants.

## 2. Materials and Methods

### 2.1. Fibroin Preparation

*Bombyx mori* cocoon shells were prepared as follows: worm and debris were discarded and cocoons were cut in thirds and washed. In order to remove sericin coating from silk fiber, 5 g of cocoons in 2 L of a 0.02 M solution of Na_2_SO_3_ in distilled water were boiled for 30 min [18,19], then the degummed fibers were washed thoroughly. Fibers were allowed to dry in a flow cabin. Silk fibers were solubilized in 9.3 M lithium bromide for a 15% (*w*/*v*) solution of silk. Silk fibroin/LiBr solution was taken to 60 °C for 4 h in a stirrer/hot plate at 60 RPM. The solution of silk fibroin/LiBr was strained to remove the not dissolved fibers. Lithium bromide was removed in a stirred cell for 7 h (Stirred Cell Model 8010, Amicon, Merck KGaA, Darmstadt, Germany) using a membrane disc of ultracel regenerated cellulose of 100 kDa (Merck KGaA, Darmstadt, Germany).

### 2.2. Estimation of Fibroin Concentration

Fibroin concentration was quantified by Lowry’s method using the Kit of Dc Protein Assay Bio-Rad. A total of 10 μL of sample was tested in quadruplicate and then 50 μL of Reagent A+S and 400 μL of Reagent B were added. They were homogenized and incubated for 15 min at room temperature. Absorbance was quantified at 750 nm utilizing an ELISA microplate reader. Concentration was calculated from the absorbance concentration curve from known concentrations of bovine serum albumin (1.95, 1.268, 0.823, 0.535, 0.348, and 0.226 μg/μL). Assays were performed in quadruplicate and expressed in μg of protein per μL.

#### Electrophoresis Assay

Different concentrations of fibroin were evaluated in quadruplicate on polyacrylamide gel at 8% and 90 volts in an electrophoresis chamber. The gel was incubated in Coomassie blue solution overnight at room temperature under constant agitation. Gels were immediately scanned after the end of the protocol and visualized to be quantified with ImageJ.

### 2.3. Fibroin/PEO Scaffold Preparation

In order to obtain the required viscosity for electrospinning, 2 and 2.5 wt% PEO (900 kDa, Sigma) solutions were blended into fibroin at 0.5, 1 and 1.5 wt% (Table 1). Solutions were 7.5–8 pH. The solution of PEO–fibroin was poured in a 10 mL syringe of a commercial electrospinning instrument (Standard unit, NEU-01, Kato tech Co., Ltd., Kyoto, Japan). An electrical field of 10 kV was applied to the PEO–fibroin blend to attain a stable Taylor cone. A syringe pump fed the solution into the needle at 1 mL/h. An electrospun fibrous mat was collected on circular cover glasses of 20 mm diameter attached to a grounded 10 × 15 cm aluminum plate, located 10 cm from the needle at 25 °C. The x-axial sliding device moved the base of the needle at 2.5 RPS.

### 2.4. Characterization of Fiber Morphology

The morphology of the electrospun scaffolds of PEO–fibroin were characterized using high-resolution scanning electron microscopy (HRSEM-AURIGA, Zeiss, Oberkochen, Germany) at 2 kV. The samples were analyzed at 2500, 5000, 7000 and 9000 times magnification. A total of 200 fibers and pores were measured at random on images of magnification at 2500X using the ImageJ software to obtain the mean superficial pore size, fiber diameter, and standard deviation.

### 2.5. Contact Angle Measurement

The contact angle of a drop of distilled water on the scaffolds surfaces was measured enabling a comparison to be made. Measurements were taken using a contact angle micrometer (Tantec Inc., Lunderskov, Kolding, Denmark) as follows: Place the scaffolds on the holder, then turn the intensity up to ¾ power so the image can be projected on the screen. Move the sample stage to the measure position (the scaffold should be aligned with the horizontal cross-line). Later, locate the scaffold surface precisely under the syringe needle and release a drop of 10 μL. Bring the specimen holder up until the droplet touches the surface and complete the transfer of the droplet. Wait for the droplet to stabilize and adjust the droplet projection until its left edge touches the vertical cross-line. Finally, rotate the protractor with a hairline on the measuring screen until the hairline crosses the droplet apex and read the contact angle on the scale. Measurements of 9 droplets per sample were taken at a temperature of 20 °C.

### 2.6. Scaffold Properties by ATR-FTIR Spectroscopy and X-Ray Diffraction

With the aim of observing the functional groups of PEO and fibroin incorporated in the matrices, the electrospun scaffolds were measured by attenuated total reflectance Fourier-transform infrared (ATR-FTIR) spectroscopy (VARIAN 640-IR, Varian Inc., Palo Alto, CA, USA). X-ray diffraction was performed using D8 Advance x-ray (Bruker, Billerica, MA, USA) diffractometer with a copper source (1.541 Å).

### 2.7. Cell Culture

Human epithelial cell line (HaCaT) were maintained in Dulbecco’s Modified Eagle’s Medium (DMEM; 12800-017, Gibco, Toronto, ON, Canada) containing 1% L-glutamine, 10% fetal bovine serum (FBS), 100 U/mL penicillin, 100 μg/mL streptomycin in 5% CO_2_ at 37 °C. Sterilized cover glasses of 20 mm diameter coated with the electrospun silk PEO–fibroin were placed on each of the wells of a 12-well-plate. HaCaT cultures from passage 2 at a concentration of 15 x 105 mL were seeded on the scaffolds and grown to 80% confluence in specific medium supplemented with 10% FBS. In advance of the assays, the cells were starved for 12 h to take them to the G0/G1 phase with medium supplemented with 2% FBS. Afterwards, cells were rinsed with phosphate buffered saline (PBS) and incubated with fresh culture medium to be grow on the scaffolds for 48 and 72 h. The group without scaffold was considered negative control.

### 2.8. Cell Viability Assays

Cytotoxicity and viability of HaCaT cells were studied by colorimetric estimation using 3-(4, 5-dimethylthiazol-2-yl)-2, 5-diphenyltetrazolium bromide (MTT, M6494, Thermo Fisher Scientific, Waltham, MA, USA) assay. The amount of formazan was directly proportional to the number of viable cells. After its growth on scaffolds at 48 and 72 h, ELISA plates with each treatment group were washed with 1X PBS and then incubated for 3 h in fresh medium containing MTT (0.5 mg/mL) at 37 °C. Later, the MTT-containing medium was discarded, and the cells were incubated in dimethyl sulfoxide. The intensity of the product was read at 570 nm using an ELISA microplate reader. Photographs in clear field at a magnification of 20X were acquired with a high-resolution camera attached to an optical microscope. All the experiments were performed in triplicate, using the well-plate as a control.

### 2.9. Evaluation of Cell Morphology

Fluorescence staining of the cytoskeleton (actin) and cell nucleus were performed by double staining [20]. After incubation times, cells were fixed with 4% paraformaldehyde at room temperature. The plate was covered with aluminum foil to agitate gently in a shaker for 15 min. Later cells were permeabilized using 0.1% Triton X-100. Cells were rinsed and stained using DAPI (CAS 28718-90-3, Sigma-Aldrich, Darmstadt, Germany) at a 1:8000 dilution for 5 min in the dark. Cells were washed, and the second fluorescent label phalloidin (Thermo Fischer, CAS A12379, Waltham, MA, USA) was added at 1:100 concentration. Cells were washed and mounted with Vectashield mounting media and observed under a confocal microscope. The number of viable nuclei within the zone of interest was quantified with respect to the control using ImageJ software.

### 2.10. Statistical Analysis

The Prism v.7.0 software (GraphPad Software, Inc., San Diego, CA, USA) was used for statistical analysis, applying one-way ANOVA and Tukey’s multiple comparison tests. The difference was regarded as statistically significant when the *p*-value was less than 0.05 (* *p* < 0.05, ** *p* < 0.01, and *** *p* < 0.001).

## 3. Results

### 3.1. Estimation of Fibroin Concentration

**Figure 1:**
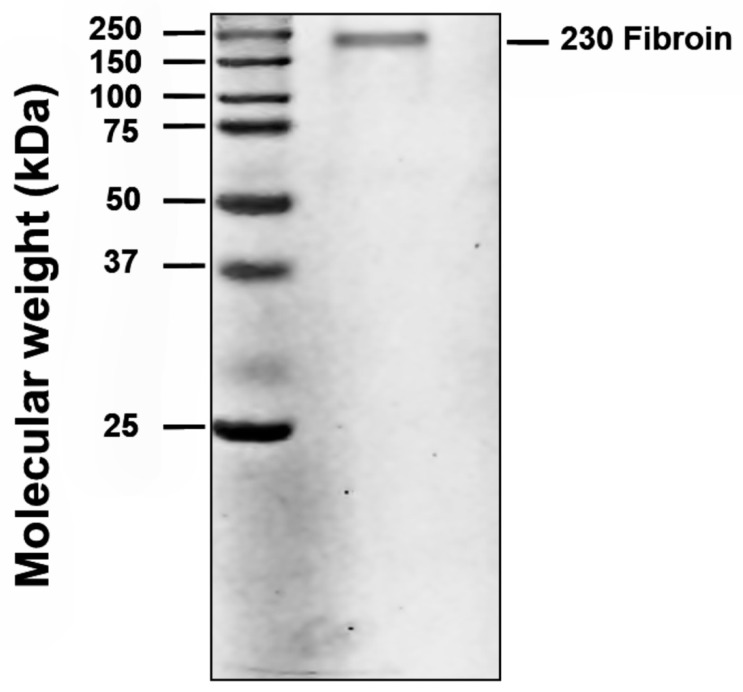
The molecular weight of purified *Bombyx mori* silk fibroin as displayed by gel electrophoresis.

Fibroin was extracted from *Bombyx mori* cocoon shells and desalted by ultrafiltration which reduced the desalting time up to 90% in comparison to the dialysis methods. The molecular weight of purified *Bombyx mori* silk fibroin was approximately 230 kDa (Figure 1).

### 3.2. Characterization of Fiber Morphology

Six different groups of PEO–fibroin electrospun scaffolds were prepared to evaluate their morphology effect on cell viability in order to be applied as a potential scaffold for tissue regeneration as they mimic the structure of the extracellular matrix (ECM). Table 1 shows the concentration of PEO–fibroin scaffolds and their effect on fiber diameter and superficial pore.

**Figure 2:**
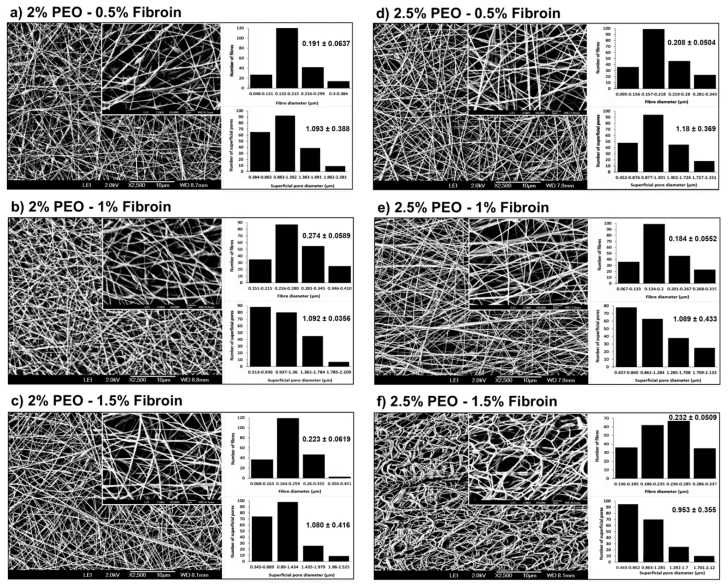
SEM micrographs of electrospun fibers, bar graphs of the fiber diameter (μm), and the superficial pore diameter (μm) at (**a**) 2% PEO–0.5% fibroin, (**b**) 2% PEO–1% fibroin, (**c**) 2% PEO–1.5% fibroin, (**d**) 2.5% PEO–0.5% fibroin, (**e**) 2.5% PEO–1% fibroin, and (**f**) 2.5% PEO–1.5% fibroin blends. Results are expressed as the mean ± standard deviation of 200 measurements taken from SEM micrographs.

Figure 2 shows typical SEM images of PEO–fibroin scaffold samples, where all matrices were uniformly distributed. The mats of 2 wt% PEO–(0.5 and 1 wt%) fibroin exhibited few beads because the high viscosities required higher voltages to break the superficial tension, so the jet was intermittent and beads were created [21]. However, the mats of (2 and 2.5 wt%) PEO–(1.5, 0.5, and 1 wt%) fibroin showed fibers without beads. Increasing the concentration of fibroin in the (2 and 2.5 wt%) PEO solution, increased the viscosity enough to maintain a stable jet to obtain uniform fibers [22]. The mat of 2.5 wt% PEO and 1.5 wt% fibroin did not show beadings and presented the morphology of moist noodles due to the effect of the solvent [23]. When the temperature of the system and the voltage was not enough, the solvents tended to have a slow evaporation and the remaining solvent in the fibers (these conditions could be the cause of a morphology of moist noodles) had to be eliminated by a dry process (35 °C for 1 h under vacuum conditions). All PEO–fibroin electrospun scaffolds showed a 3-D fibrous mesh that could provide a network where cells move and interact with themselves and the scaffold. Table 1 shows the concentration of PEO–fibroin scaffolds and their effect on fiber diameter and superficial pore. The mean diameter and superficial pore average diameter resulting from measuring 200 fibers and pores at random is shown in Figure 2.

### 3.3. Contact Angle Measurement

Table 2 shows the average of the contact angles of 9 droplets of each of the six scaffolds and the breast implant without scaffold. The PEO–fibroin scaffolds increased up to 79% the hydrophilicity of the surfaces when compared to the surface of the implant.

### 3.4. Scaffold Properties by ATR-FTIR Spectroscopy and X-Ray Diffraction

**Figure 3:**
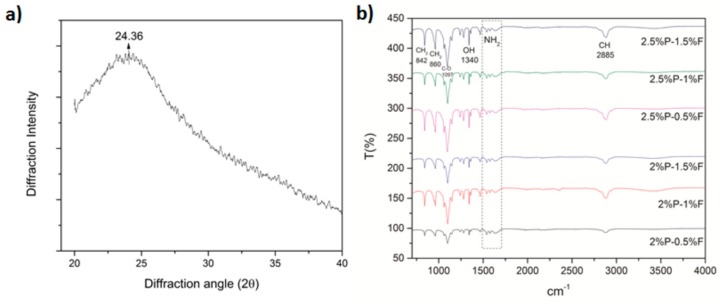
(**a**) Diffraction pattern of silk fibroin; (**b**) FTIR Spectra of PEO–fibroin electrospun scaffolds.

Figure 3a shows the diffraction pattern of silk fibroin with a peak at 24.36° (d = 3.66 Å, medium strong) indicating that silk fibroin contains high β-sheet components [24,25]. FTIR spectra observed on all PEO–fibroin blends displayed absorption bands around 840, 960, and 1467 cm^−1^ corresponded to CH_2_; 1097 cm^−1^, corresponded to C–O; 1340 cm^−1^, corresponded to OH and 2883 cm^−1^, corresponded to CH which were attributed to PEO [26]. Figure 3b shows the typical bands of amino groups corresponding to fibroin that can be distinguished at wavenumbers 1538, 1575, and 1629 cm^−1^ [27].

### 3.5. Cell Viability

**Figure 4:**
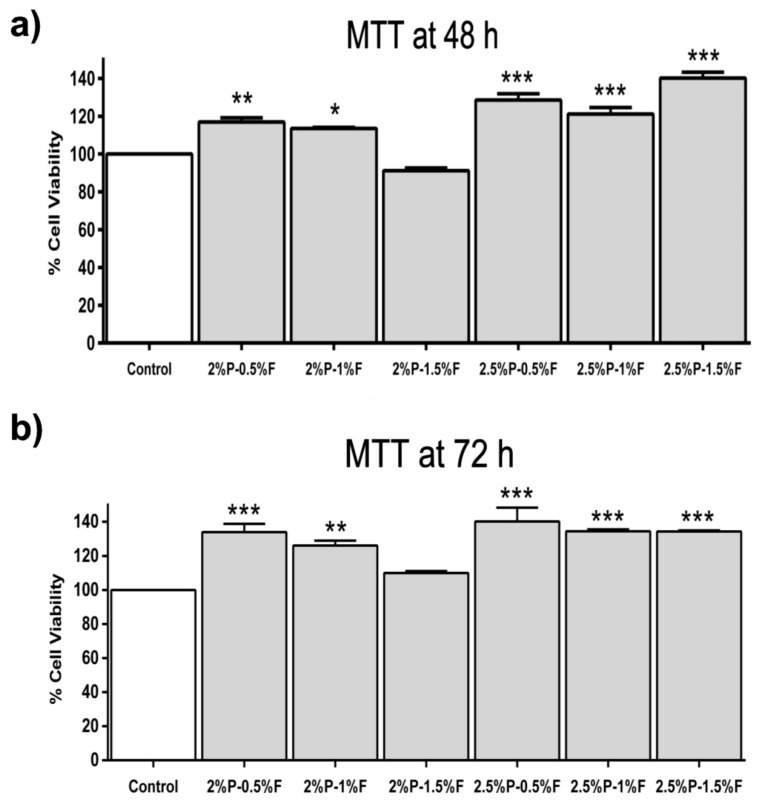
MTT assay at (**a**) 48 h and (**b**) 72 h. Results are expressed as the mean ± standard deviation of triplicates. *p*-value less than 0.05 (* *p* < 0.05, ** *p* < 0.01, and *** *p* < 0.001) indicates statistically significant difference by one-way ANOVA and Tukey’s test.

The reduction activity of the methyl thiazolyl tetrazolium (MTT) was examined after 48 and 72 h of seeding HaCaT cells on electrospun PEO–fibroin scaffolds to the measurement of cell viability. The results in Figure 4a,b reveal HaCaT cells seeded on PEO–fibroin scaffolds showed significantly (*p* < 0.001) greater cell viability in all evaluated conditions when compared with the biocompatibility of the control after 48 and 72 h.

### 3.6. Evaluation of Cell Morphology

**Figure 5:**
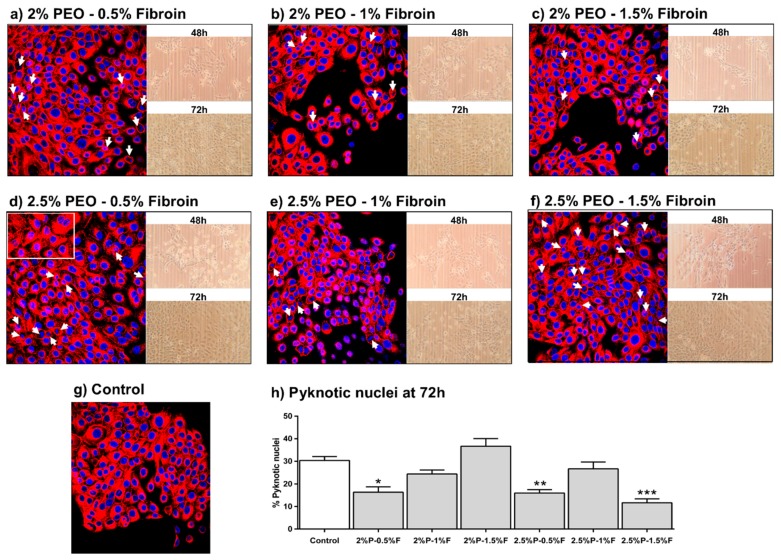
HaCaT cells seeded on PEO–fibroin electrospun mats after 72 h. (**a**–**f**) Fluorescence staining of nucleus (blue), f-actin of cytoskeleton (red), and phase contrast photographs; (**h**) % pyknotic nuclei quantification at 72 h. Results are expressed as the mean ± standard deviation of triplicates. *p*-value less than 0.05 (* *p* < 0.05, ** *p* < 0.01, and *** *p* < 0.001) indicates statistically significant difference by one-way ANOVA and Tukey’s test.

The effect of electrospun PEO–fibroin on cytoskeleton organization was studied by fluorescence microscopy of HaCaT cells in which f-actin filaments and nuclei were stained with a fluorescent dye. Figure 5 shows f-actin filaments generally located at the cell periphery of cells seeded onto 2%P–0.5%F, 2%P–1%F, 2.5%P–0.5%F, 2.5%P–1%F, and 2.5%P–1.5%F (pyknotic cells indicated by arrows).

## 4. Discussion

In this study, we examined the potential of electrospun PEO–fibroin scaffold as regeneration of HaCaT cells [28]. Fibroin was extracted from *Bombyx mori* cocoon shells and blended with PEO to be later electrospun on cover glasses. The use of PEO added stability and mechanical support to the scaffold, while fibroin provided biological cues to the cells.

SEM micrographs showed a scaffold composed of 0.25 µm diameter fibers and 0.76 µm size superficial pores. This morphology, demonstrated in a previous study of our laboratory, provides high structural properties due to its high elastic modulus and elastic behavior to breast implants under a puncture tensile test [29]. Silk fibroin showed that at different concentrations has an effect in the network conformations of PEO scaffolds modifying the microstructure. Thus, fibroin incorporation influences the regulation of cell adhesion and proliferation of keratinocytes cells. This approach could facilitate the manipulation of addition of silk fibroin in the scaffolds to be electrospun and obtain fibers without defects such as beads and provide biocompatible surfaces [30,31,32,33].

From the contact angle measurement results, it was clear that when coating the implants with the PEO–fibroin scaffolds, the hydrophilicity of their surfaces was increased from 58% to 79% when compared to the contact angle of the uncoated implant. Therefore, the increased concentration of PEO and fibroin may have a major influence in the increase of hydrophilicity of the scaffolds.

Moreover, the biological functionality of the natural polymer silk fibroin appeared to enhance the HaCaT viability (p < 0.001), as assessed by MTT assay, where all blends showed better viability and stable behavior at 48 and 72 h when compared with the control, particularly the 2.5% PEO–0.5% fibroin blend as see on Figure 4. This was attributed to the interactions between fibroin and HaCaT cells as it was observed on the pyknotic nuclei assay on Figure 5 where the 2% PEO–0.5% fibroin, 2.5% PEO–0.5% fibroin, and 2.5% PEO–1.5% fibroin samples were shown to improve cell attachment. Actin participated highly in protein–protein interactions and also, a large number of actin-binding proteins made actin a critical player in cell motility and the maintenance of cell shape and polarity [34]. Consistent with Yan [35], the fiber scaffolds provided a more advantageous microenvironment than the control.

This study demonstrated that biocompatibility and physical characteristics of substrates were improved by surface modification with electrospun PEO–fibroin fibrous scaffolds. For instance, randomly oriented fibers showed to improve cell viability, in agreement with Kasoju [36], as tested with MTT assay. The blend of 2.5% PEO–1.5% fibroin showed the best results of viability and less percentage of pyknotic nucleus which demonstrated less direct impact on cell damage. We observed that the mesh morphology at this blend was different to the others providing a better scaffold to promote cell adhesion and proliferation. It seemed that mesh morphology had a direct impact on cell damage.

These results demonstrate the possibility of changing chemistry and physical behavior by coating the surface of biomaterials with electrospun PEO–fibroin. The PEO–fibroin scaffold was designed so that HaCaT cells could achieve viability at this microenvironment. This can have the ability to regulate cell behavior from attachment, but further investigation needs to carry on testing cell adhesion genes. In addition, an evaluation to define if the distribution of silk fibroin changes with the variation of the ratio of PEO and silk fibroin, and if this influences the cell viability of the prepared electrospun scaffolds by chemical synthesis systems [37,38], might prove an important area for future research.

## 5. Conclusions

Effectiveness of electrospun artificial and natural polymers (PEO and silk fibroin) on breast implants was tested on HaCaT cells. Fibroin, extracted from *Bombyx mori* cocoons, provided biological cues to HaCaT cells to adhere and proliferate, while PEO added stability and mechanical support to the scaffold. These proteins showed diverse network conformations at different combinations, so modification of the microstructure of the scaffold, cell adhesion, and proliferation could be regulated. Further investigation needs to carry on testing cell adhesion genes. Furthermore, these proteins can be used to fabricate scaffolds for more specific applications in the biomedical engineering area.

## Figures and Tables

**Table 1:** Silk fibroin-polyethylene oxide (PEO)—blends and scaffold measurements.

PEO Concentration (wt%)	Silk Fibroin Concentration (wt%)	Average Fiber Diameter (µm)	Average Superficial Pore (µm)
2	0.5	0.191 ± 0.0637	1.093 ± 0.388
	1	0.274 ± 0.0589	1.092 ± 0.356
	1.5	0.223 ± 0.0619	1.080 ± 0.416
2.5	0.5	0.208 ± 0.0504	1.180 ± 0.369
	1	0.184 ± 0.0552	1.089 ± 0.433
	1.5	0.232 ± 0.0509	0.953 ± 0.355

**Table 2:** Contact angle of the PEO–fibroin scaffolds and a breast implant surface.

Implant without Scaffold	2% PEO–0.5% Fibroin	2% PEO–1% Fibroin	2% PEO–1.5% Fibroin	2.5% PEO–0.5% Fibroin	2.5% PEO–1% Fibroin	2.5% PEO–1.5% Fibroin
115.6 ± 0.6°	48.3 ± 2.4°	44.5 ± 2°	40 ± 1°	31.3 ± 2.3°	27 ± 2.6°	24.7 ± 2°
